# Donor funding health policy and systems research in low- and middle-income countries: how much, from where and to whom

**DOI:** 10.1186/s12961-017-0224-6

**Published:** 2017-08-31

**Authors:** Karen Ann Grépin, Crossley Beth Pinkstaff, Zubin Cyrus Shroff, Abdul Ghaffar

**Affiliations:** 1 Wilfrid Laurier University, 75 University Avenue West, Waterloo, ON N2L 3C5 Canada; 2 Institute for Food Policy Research, Washington, DC USA; 3 Alliance for Health Policy and Systems Research, World Health Organization, Geneva, Switzerland

## Abstract

**Background:**

The need for sufficient and reliable funding to support health policy and systems research (HPSR) in low- and middle-income countries (LMICs) has been widely recognised. Currently, most resources to support such activities come from traditional development assistance for health (DAH) donors; however, few studies have examined the levels, trends, sources and national recipients of such support – a gap this research seeks to address.

**Method:**

Using OECD’s Creditor Reporting System database, we classified donor funding commitments using a keyword analysis of the project-level descriptions of donor supported projects to estimate total funding available for HPSR-related activities annually from bilateral and multilateral donors, as well as the Bill and Melinda Gates Foundation, to LMICs over the period 2000–2014.

**Results:**

Total commitments to HPSR-related activities have greatly increased since 2000, peaked in 2010, and have held steady since 2011. Over the entire study period (2000–2014), donors committed a total of $4 billion in funding for HPSR-related activities or an average of $266 million a year. Over the last 5 years (2010–2014), donors committed an average of $434 million a year to HPSR-related activities. Funding for HPSR is heavily concentrated, with more than 93% coming from just 10 donors and only represents approximately 2% of all donor funding for health and population projects. Countries in the sub-Saharan African region are the major recipients of HPSR funding.

**Conclusion:**

Funding for HPSR-related activities has generally increased over the study period; however, donor support to such activities represents only a small proportion of total DAH and has not grown in recent years. Donors should consider increasing the proportion of funds they allocate to support HPSR activities in order to further build the evidence base on how to build stronger health systems.

**Electronic supplementary material:**

The online version of this article (doi:10.1186/s12961-017-0224-6) contains supplementary material, which is available to authorized users.

## Background

The importance of health policy and systems research (HPSR) in providing evidence on how to best strengthen health systems has been increasingly recognised and the field has witnessed rapid development in recent years [1]. HPSR is defined by the Alliance for Health Policy and Systems Research (herein the Alliance) as the production and application of knowledge to improve how societies organise themselves in order to achieve health goals [2]. It encompasses research on how societies plan, manage and finance health services and the role and interests of different actors in the health system. It is both multi-disciplinary and inter-disciplinary in nature [3]. While its role has been well recognised in strengthening health systems, the recent Ebola epidemic highlighted the need to further invest into HPSR activities in order to generate more generalisable lessons on how to build stronger, more resilient health systems [1, 4–6].

The growth and development of the field is greatly dependent on the availability of adequate and reliable funding. Despite the calls for increased investments in HPSR in low- and middle-income countries (LMICs), resources for HPSR remain relatively modest compared to many other areas in global health [5, 7]. Further, reliable and accessible information on funding for HPSR globally is limited. Quantifying financial resources for HPSR is challenging because HPSR covers a broad set of issues and activities, many of which are only components of larger projects. Previous studies have confirmed that international donors of development assistance for health (DAH) are the major source of financial support to HPSR, but few donors break down funding for specific activities in their financial statements, making it difficult to parse out resources allocated specifically for HPSR [8]. In addition, the landscape of health donors has expanded in recent years, for whom comparable data on funding flows may not be available [9].

Tracking and understanding fund flows to support HPSR activities is critical both to inform decision-makers and to serve as the basis for future advocacy efforts. To our knowledge, this is the first comprehensive study of this topic. To date, there have been only a few studies tracking the funding for HPSR, all of which have employed surveys of institutions and actors involved in HPSR in LMICs [8, 10, 11]. Based on an extrapolation from a survey of HPSR generating institutions in LMICs, Gonzalez-Block and Mills [10] estimated annual funding for HPSR relevant to LMICs to be approximately $58 million. In a follow-up survey using a similar methodology, Bennett et al. [8] found that funding for HPSR had not grown based on a survey of HPSR funding agencies. More recently, Adam et al. [7] conducted another follow-up survey and found only modest growth in funding for HPSR in LMICs. All of these previous studies are limited by the sample of respondents, which raises questions about the representativeness of the data.

This paper builds on previous studies by conducting a systematic keyword content analysis of the descriptions of aid projects funded by bilateral and multilateral donors as well as the Bill and Melinda Gates Foundation (BMGF) over the period 2000–2014. A similar methodology has been used to characterise DAH into broad health focus areas, such as HIV/AIDS, health sector support and non-communicable diseases [12, 13]. The remainder of this paper is divided into three sections. We begin with a description of methods and data used for the study. Next, we report our findings and, finally, we discuss the implications of these findings for the field and draw overall conclusions.

## Methods

There are two main challenges to estimating funding for HPSR activities. The first is to identify projects encompassing HPSR activities from the universe of projects contained in aid databases. The second challenge arises from the fact that HPSR activities are frequently only one component of larger, often multi-sector or multi-component projects. We developed a multi-step methodology to address each of these concerns.

The primary source of data for this study was the Creditor Reporting System (CRS) database, which is the most widely used aid database and which is maintained by the Organization for Economic Co-operation and Development (OECD) [14]. The CRS aggregates annual transaction-level data on official development assistance projects supported by bilateral aid agencies from 29 countries and multilateral donors, including the World Bank, Global Alliance for Vaccines and Immunization (GAVI), and the Global Fund to Fight AIDS, Tuberculosis and Malaria (GFATM). Since 2009, the CRS also includes data from grants from the BMGF. Additional file 1: Table S1 provides a list of donors included in the CRS database. At the time of writing of this manuscript, 2014 was the most recent year for which there was complete CRS data available.

Projects in the CRS database are categorised into programmatic areas represented by sector codes. For this study, we used data from CRS sector codes 120 (health), 130 (population and reproductive health including HIV) and 160.64 (social mitigation of HIV). This approach, which has been used widely by others to define health-related aid, allows us to capture all aid that has a primary intent to improve health and population outcomes [12, 14, 15]. In addition to the sector codes, the CRS data also includes a project title, a short description and a longer description for each project, which usually contains descriptive information reported by the donor.

Each record in the CRS also includes reported annual commitments and disbursements. A commitment as a firm written obligation by a government or official agency, to provide resources of a specified amount under specified financial terms and conditions. A disbursement is the actual payment made against a previous commitment. We tracked commitments rather than disbursements because there are many factors that contribute to delays between commitments and disbursements, many of which are unrelated to donors themselves. Additionally, we feel that commitments better reflect donor priorities [16]. Other research has shown that, over the study time period, actual disbursements track well with commitments and disbursements rates for health projects has been quite high [17]. All data are reported in current 2014 US dollars.

In addition to the CRS, we also had access to data on core funding to the Alliance for HPSR activities from three current core donors. While we do not include this data in our overall trends analysis, we do include these amounts to our estimates of total support by specific donors for HPSR from 2000 to 2014.

### Content analysis

The next step was to identify projects containing HPSR activities. To do this, we developed a method to systematically categorise projects in the CRS database based on keywords in titles and descriptions of individual projects. Given that the Alliance has published extensively on the topic of HPSR, we used all flagship reports and manuals that were available in mid-2014 and available in English, French or Spanish (Additional file 2: Table S2), and used these documents to generate a list of HPSR keywords using an online application to identify the most frequent words and word combinations (word combinations could have a maximum of three non-article words). All words and word combinations with at least 10 mentions in any of the above referenced publications were then further analysed by the authors to exclude all word combinations that were not specific to HPSR activities. The final keyword list was then split into words that were specific to health policy and health system (HPHS) activities and those related to research activities (Additional file 3: Table S3). Using a python-based search algorithm, we then searched the project titles, short descriptions and long descriptions for all of the projects in the CRS database that contained both a HPHS and a research keyword, which were categorised as HPSR projects. For the purposes of also identifying donor support of overall HPHS activities, we also report on all projects that contained at least one HPHS keyword, regardless of whether it also contained a research keyword.

We also examined the content of projects that did not include any HPHS keywords (roughly 44% of all health projects) to ensure that relevant projects in the dataset were not accidentally excluded from funding estimates. When relevant projects were identified, we added additional keywords to our HPHS and HPSR keyword lists to ensure that they were not missed in repeated searches. Additionally, we noticed a number of multi-sector projects in the dataset that included funding for activities outside the health sector. We used a set of flag words for a number of topic areas (“immigration”, “development policy”, “poverty reduction”, “nutrition”, “sanitation”) to identify specific multi-sector projects such as World Bank poverty reduction support credits and structural adjustment credits. We reviewed project descriptions that contained flag words and adjusted donor commitments to these projects according to the percentage of funding allocated specifically to health using donor documentation.

### In-depth review

To verify that the identified HPSR projects were in fact capturing HPSR funding, the next step was to carry out an additional in-depth analysis for a subset of projects, including projects with the largest financial commitments from large multilateral donors (International Bank for Reconstruction and Development (IBRD), International Development Association (IDA), GAVI, GFATM) as well as the four projects with the largest financial commitments within each CRS subsector. For these projects, we searched individual donor reports, donor financial documents and funding strategy papers, and used the detailed reports to validate whether our identified projects did in fact contain significant HPSR activities.

Through our in-depth review, we found that HPHS and HPSR activities were frequent components of larger multi-sector projects or that the research component represented a portion of the total health project. This was especially the case for projects funded by multilateral agencies. Therefore, using the detailed reports, we developed a set of assumptions for a subset of donors (IDA, IBRD, GFATM, GAVI, USAID, Australian Agency for International Development, Department for International Development, and Canadian International Development Agency) to more accurately estimate the fraction of total funding for each project specifically allocated to HPSR activities. Additional file 4: Table S4 summarises the assumptions applied and methods used for each donor. All estimates of funding for HPHS and HPSR provided below reflect these donor-specific assumptions and should be interpreted as funding in support of HPSR-related activities.

## Results

Table 1 summarises aggregate funding about projects in the CRS database. Of the 332,952 Health and Population (‘health’) projects identified in the CRS database from 2000 to 2014, 186,681 projects (56% of all health projects) contained keywords relevant to HPHS activities, among which 69,187 projects (21% of all health projects) also included at least one research keyword and were thus classified as HPSR projects. Over the period 2000–2014, donor commitments to the health and population sectors exceeded $246 billion. Sixteen percent ($39.8 billion) of total commitments went to projects that included health policy and health systems keywords but only 2% of total commitments ($4 billion) were directed to HPSR-related activities.

**Table 1:** Number and value of projects for health, health policy and health systems (HPHS), and health policy and system research (HPSR)

Year	Number of projects			Value of projects (in 2014 $M USD)		
	Health	HPHS	HPSR	Health	HPHS	HPSR
2000	6483	3458	1088	5083.72	882.11	75.66
2001	10,814	5479	2184	5413.94	939.58	113.39
2002	10,962	5329	2458	5929.01	796.09	79.39
2003	14,452	6810	2534	9624.01	1388.96	132.97
2004	13,727	6617	1930	9346.93	1228.97	100.54
2005	16,842	7401	2409	11840.82	1764.99	156.83
2006	20,799	8798	3220	15407.86	2142.77	242.79
2007	23,764	13,014	3604	17055.26	2624.21	169.16
2008	25,133	13,876	4292	19971.02	3112.20	251.38
2009	32,135	19,496	6485	23677.78	4206.18	504.44
2010	30,990	18,783	8096	23638.56	3812.31	540.51
2011	32,048	20,129	8380	23038.13	3590.27	392.88
2012	31,101	17,316	7389	23218.18	3861.19	384.77
2013	31,270	19,906	6799	26903.25	4545.31	440.03
2014	32,432	20,269	8319	25864.86	4895.30	410.58
Total	332,952	186,681	69,187	$246,013	$39,790	$3995
% of all health	100%	56%	21%	100%	16%	2%
Source: Creditor Reporting System						
	Average funding per project per year			$738,885	$213,147	$57,747
	Average funding per year, in 2014 $M USD (2000–2014)			$16,401	$2653	$266
	Average funding per year, in 2014 $M USD (2010–2014)			$24,533	$4141	$434

Table 2 summarises the top donors to HPSR-related activities. There was a very high level of concentration among donors with most of the aid for HPSR coming from just a handful of donors – the United States and the Global Fund alone accounted for approximately half of all funding to support HPSR activities. The top 10 donors (United States, Global Fund, BMGF, IBRD, IDA, Canada, United Kingdom, Norway, Australia and France) accounted for 93% of total commitments to HPSR projects from 2000 to 2014.

**Table 2:** Top donors of health policy and systems research (HPSR) funding, 2000–2014

Donor		Total commitments to health (2014 $M USD)	Total commitments to HPSR (2014 $M USD)
United States		72,438.7	1262.6
Global Fund		29,317.9	574.9
Bill & Melinda Gates Foundation		10,357.7	491.7
International Bank for Reconstruction and Development		13,095.1	466.1
International Development Association		15,736.3	428.2
Canada		6327.8	214.9
United Kingdom^a^		15,180.9	123.2
Norway^a^		3577.4	110.2
Australia		3956.5	39.4
France		4038.2	37.3
Sweden^a^		2853.3	32.1
UNFPA		3951.5	28.5
Inter-American Development Bank		4319.9	25.2
EU Institutions		8192.3	22.8
UNAIDS		2482.8	22.7
Ireland		1614.6	17.4
Global Alliance for Vaccines and Immunization		7521.2	17.4
Islamic Development Bank		1815.5	17.2
Germany		5729.9	16.4
Belgium		2138.8	16.1
	Total, top 10 donors	174,026.4	3748.5
	Total, all donors	246,013.3	3995.3
	% top 10 donors/all donors	71%	94%

^a^Estimates for these three donors also include core contributions made to the Alliance in support of HPSR activities

**Fig. 1:**
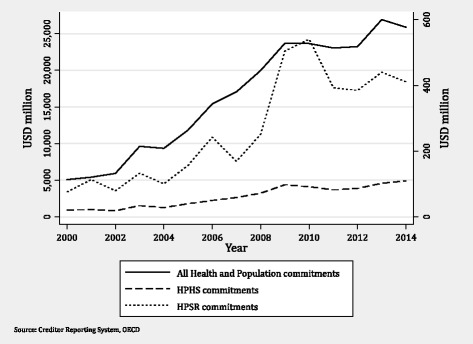
Trends in commitments for all donor funding of projects

Figure 1 graphs the trends in HPSR funding. Total health and population funding and HPHS funding are charted using the left-hand axis, while HPSR funding uses the right-hand axis. While there were steady increases in both total health aid and all HPHS aid from 2000 to 2014, aid for HPSR was more variable. Total HPSR funding was less than $100 million a year in 2000, peaked at about $540 million in 2010, and then remained at approximately $400 million a year through 2014.

**Fig. 2:**
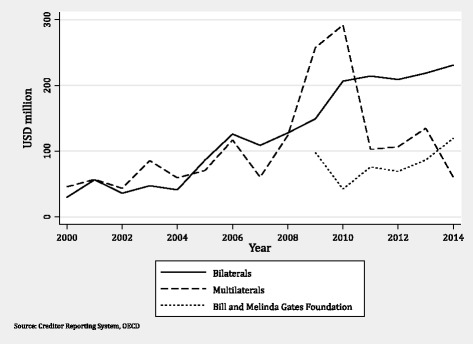
Trends in health policy and systems research funding by donor type, 2000–2014

Figure 2 disaggregates commitments for HPSR according to donor type. We show data for the BMGF only post-2009, the year they began reporting to the CRS. Until 2008, bilateral and multilateral donors provided approximately the same amount of aid for HPSR. In 2009, there was a large jump in funding from multilateral donors. This jump was due to increased aid from the IBRD in response to the economic crisis and then declined substantially (over 50%) until 2012, when it began to increase again. This increase in financial support by the IBRD has also been documented elsewhere [18]. After 2010, bilateral donors become the largest donors of funding for HPSR.

**Fig. 3:**
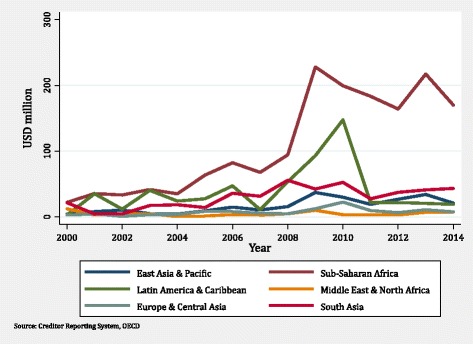
Trends in health policy and systems research funding by recipient region, 2000–2014

Figure 3 summarises patterns in the regional allocations of funding for HPSR-related activities. Countries in sub-Saharan Africa received the largest share of aid for HPSR, or roughly half of all HPSR in later years. Funding for this region doubled between 2008 and 2009. Latin American countries also demonstrated dramatic increases in funding in 2010, which corresponded to the global economic crisis. All other regions demonstrated relatively stable aid flows.

## Discussion

Using the CRS database, we tracked donor resources in support of HPSR-related activities in LMICs. We found donors committed almost $4 billion to these projects from 2000 to 2014 or, on average, $266 million a year over the entire period. More recently, donors committed, on average, $434 million a year from 2010 to 2014. However, these figures represented only approximately 2% of total donor support to health and population programmes over the entire study period. Further, while there was a gradual increase in funding over the entire study period, new funding commitments have been relatively flat since 2011. Aggregate figures also mask the important shifts in funding from bilateral to multilateral donors and across recipient country regions during the study period.

Our analysis also highlights the extent of donor concentration that exists in HPSR funding. We found that, while most major DAH donors contributed funding to HPSR projects, 93% of this funding came from just 10 donors. The various agencies of the United States government and the Global Fund alone accounted for approximately half of all funding for HPSR funding. This finding suggests that HPSR has a relatively narrow funding base and may be vulnerable should the priorities of key donors shift from HPSR in the future.

The largest share of funding for HPSR is committed to countries in sub-Saharan Africa, which is appropriate given the region’s burden of disease as well as the difficulty that very low-income countries potentially have in raising domestic resources for HPSR. In the interest of sustainability and increased national ownership of HPSR, it is important to ensure that at least some of this funding is used to strengthen national researcher capacity as well as sensitise decision-makers to the potential of HPSR to inform improved national policy. Our analysis did not allow an assessment on how these resources were being spent within countries.

Finally, while funding for HPSR-related activities has increased, it is important to view this in perspective of the quantum of funding available for health research. In 2014, the National Institutes of Health in the United States of America, invested close to $30 billion in medical research, an amount close to 70 times our estimate for HPSR funding in that same year [19]. Given the central role that HPSR has in enabling the strengthening of health systems and improving health, donors should consider increasing the share of aid allocated to HPSR activities and there is a need to diversify the funding base for HPSR-related activities.

There are some limitations to our study. First, we only tracked funding provided by traditional global health donors that report their aid activities to the CRS. Not all donor activities are reported, although previous research does suggest that the majority of financial support for HPSR activities does come from traditional aid donors. However, as middle-income countries increasingly support HPSR through their domestic resources and have even emerged as donors in some instances [20], it is important that these new funding sources are reflected in standardised databases such as the CRS. We also limited ourselves to projects with CRS sector codes for health and population, but it is possible that other projects not identified in this way may also support HPSR activities.

Second, using our approach, we can only identify projects that have HPSR activities in the titles or long descriptions and we cannot determine the fraction of the total resources in a project that are actually spent on research activities. Our secondary in-depth analysis of donor documents was designed to try to better account for this, but it is based on a set of donor-specific assumptions. Third, while most projects provide a short description in English, not all documents are reported in English. We were able to translate some of the keywords into French and Spanish and include these in our keyword searches; however, funding from donors who report in other languages may be underreported.

Fourth, we have tracked commitments, a measure which we believe better represents the intent of donors compared to disbursements, but it does lead to greater fluctuations in the data year to year, in particular if multiyear grants are announced in a given year. In addition, since not all commitments are fully disbursed to countries, this also may represent an overestimate of funding for HPSR-related activities. Finally, our research method relies heavily on the existence of specific keywords to identify HPSR activities and on the fact that all donors would use these terms to report their aid activities. Efforts are needed to standardise terminologies around HPSR, an area where the Alliance could play a convening role, bringing together donors and those who maintain aid databases to better understand and harmonise donor-reporting mechanisms.

## Conclusions

There is a growing consensus that strong, responsive and resilient health systems are key to health improvements globally and that HPSR can greatly help to build such systems. While there have been significant increases in donor support to HPSR-related activities since the early 2000s, funding levels have been relatively stagnant over the past few years, they are heavily concentrated among a few donors, and only represent a small fraction of total development assistance for health. Continuing to grow the evidence base on how to build and sustain these health systems will continue to require sufficient and stable funds for HPSR. By developing a new approach to track donor resources for HPSR that uses publically available databases and that can be easily updated, this paper has provided the first long-term estimates of financial support of HPSR-related activities from traditional health donors that can be useful for both monitoring and future advocacy purposes.

## Additional files

Additional file 1: Table S1. List of donors in the OECD Creditor Reporting System Database, 2000–2014. (PDF 41 kb)

Additional file 2: Table S2. Alliance reports searched for keyword analysis. (PDF 35 kb)

Additional file 3: Table S3. List of keywords used in analysis. (PDF 39 kb)

Additional file 4: Table S4. Donor-specific assumptions for in-depth project review. (PDF 40 kb)

## Abbreviations

BMGF: Bill and Melinda Gates Foundation
CRS: Creditor Reporting System
DAH: development assistance for health
GAVI: Global Alliance for Vaccines and Immunization
GFATM: Global Fund to Fight AIDS, Tuberculosis and Malaria
HPHS: health policy and health system
HPSR: health policy and systems research
IBRD: International Bank for Reconstruction and Development
IDA: International Development Association
LMICs: low- and middle-income countries
OECD: Organization for Economic Co-operation and Development
USAID: United States Agency for International Development

## References

[1] Sheikh K, Gilson L, Agyepong IA, Hanson K, Ssengooba F, Bennett S (2011). Building the field of health policy and systems research: framing the questions. PLoS Med.

[2] World Health Organization. What is Health Policy and Systems Research and Why Does it Matter? 2007. http://www.who.int/alliance-hpsr/about/hpsr/en/. Accessed 28 Oct 2015.

[3] Gilson L, Hanson K, Sheikh K, Agyepong IA, Ssengooba F, Bennett S (2011). Building the field of health policy and systems research: social science matters. PLoS Med.

[4] World Health Organization. Changing Mindsets. 2012. http://www.who.int/alliance-hpsr/whostrategyhpsr/en/. Accessed 28 Oct 2015.

[5] Ghaffar A, Tran N, Røttingen J-A, Kieny M-P (2014). Health policy and systems research: building momentum and community. Bull World Health Organ.

[6] Kruk ME, Myers M, Varpilah ST, Dahn BT (2015). What is a resilient health system? Lessons from Ebola. Lancet.

[7] Adam T, Ahmad S, Bigdeli M, Ghaffar A, Røttingen J-A. Trends in health policy and systems research over the past decade: still too little capacity in low-income countries. Noor AM, editor. PLoS ONE. 2011;6(11):e27263. 10.1371/journal.pone.0027263 PMC3222652 22132094

[8] Bennett S, Adam T, Zarowsky C, Tangcharoensathien V, Ranson K, Evans T (2008). From Mexico to Mali: progress in health policy and systems research. Lancet.

[9] Ravishankar N, Gubbins P, Cooley RJ, Leach-Kemon K, Michaud CM, Jamison DT (2009). Financing of global health: tracking development assistance for health from 1990 to 2007. Lancet.

[10] Gonzalez Block MA, Mills A (2003). Assessing capacity for health policy and systems research in low and middle income countries*. Health Res Policy Syst.

[11] Alliance for Health Policy and Systems Research. Strengthening Heath Systems: the Role and Promise of Policy and Systems Research. 2004. http://www.who.int/alliance-hpsr/resources/Strengthening_complet.pdf. Accessed 20 Oct 2016.

[12] Powell-Jackson T, Borghi J, Mueller DH, Patouillard E, Mills A (2006). Countdown to 2015: tracking donor assistance to maternal, newborn, and child health. Lancet.

[13] Dieleman JL, Graves C, Johnson E, Templin T, Birger M, Hamavid H (2015). Sources and focus of health development assistance, 1990–2014. JAMA.

[14] Grépin KA, Leach-Kemon K, Schneider M, Sridhar D (2012). How to do (or not to do) … Tracking data on development assistance for health. Health Policy Plan.

[15] Grépin KA (2012). HIV donor funding has both boosted and curbed the delivery of different non-HIV health services in sub-Saharan Africa. Health Aff.

[16] Sridhar D, Batniji R (2008). Misfinancing global health: a case for transparency in disbursements and decision making. Lancet.

[17] Institute for Health Metrics and Evaluation. Financing Global Health 2015: Development assistance steady on the path to new Global Goals. http://www.healthdata.org/policy-report/financing-global-health-2015-development-assistance-steady-path-new-global-goals. Accessed 12 June 2017.

[18] Leach-Kemon K, Chou DP, Schneider MT, Tardif A, Dieleman JL, Brooks BPC (2012). The global financial crisis has led to a slowdown in growth of funding to improve health in many developing countries. Health Affairs (Project HOPE).

[19] National Institutes of Health. Budget. 2014. http://www.nih.gov/about-nih/what-we-do/budget. Accessed 25 Jan 2016.

[20] Fan VY, Grépin KA, Shen GC, Chen L (2014). Tracking the flow of health aid from BRICS countries. Bull World Health Organ.

