# Supplementary material for: Donor funding health policy and systems research in low- and middle-income countries: how much, from where and to whom
Source: Health Res Policy Syst. 2017 Aug 31;15:68. doi: 10.1186/s12961-017-0224-6 (PMC5577666; doi:10.1186/s12961-017-0224-6)
Supplement: Supplementary file 1 — List of donors in the OECD Creditor Reporting System Database, 2000–2014. (PDF 41 kb) [file 12961_2017_224_MOESM1_ESM.pdf]

Appendix Table 1: List of donors in the OECD CRS Database, 2000-14

Bilateral donors		Multilateral donors		Private donors	
1	Algeria	1	AfDB	1	Bill & Melinda Gates Foundation
2	Australia	2	AfDF		
3	Austria	3	Arab Fund (AFESD)		
4	Belgium	4	AsDB		
5	Bulgaria	5	AsDB Special Funds		
6	Canada	6	BADEA		
7	Chinese Taipei	7	CarDB		
8	Croatia	8	Climate Investment Fund		
9	Cyprus	9	EBRD		
10	Czech Republic	10	GAVI		
11	Denmark	11	GEF		
12	Estonia	12	Global Fund		
13	EU Institutions	13	Global Green Growth Institute		
14	Finland	14	IAEA		
15	France	15	IBRD		
16	Germany	16	IDA		
17	Greece	17	IDB		
18	Hungary	18	IDB Sp.Fund		
19	Iceland	19	IFAD		
20	Iraq	20	IFC		
21	Ireland	21	IMF		
22	Israel	22	IMF (Concessional Trust Funds)		
23	Italy	23	Isl.Dev Bank		
24	Japan	24	Montreal Protocol		
25	Kazakhstan	25	Nordic Dev.Fund		
26	Korea	26	OFID		
27	Kuwait (KFAED)	27	OSCE		
28	Latvia	28	UN Agencies		
29	Libya	29	UNAIDS		
30	Liechtenstein	30	UNDP		
31	Lithuania	31	UNECE		
32	Luxembourg	32	UNEP		
33	Malta	33	UNFPA		
34	Netherlands	34	UNHCR		
35	New Zealand	35	UNICEF		
36	Norway	36	UNPBF		
37	Poland	37	UNRWA		
38	Portugal	38	UNTA		
39	Qatar	39	WFP		
40	Romania	40	WHO		
41	Russia				
42	Saudi Arabia				
43	Slovak Republic				
44	Slovenia				
45	Spain				
46	Sweden				
47	Switzerland				
48	Thailand				
49	Turkey				
50	United Arab Emirates				
51	United Kingdom				
52	United States				

Note: AfDB=African Development Bank, AfDF=African Development Fund, AsDB=Asian Development Bank, BADEA=Arab Bank for Economic Development in Africa, EBRD=European Bank for Reconstruction and Development, GAVI=The Vaccine Alliance, GEF=Global Environment Facility, IBRD=International Bank for Reconstruction and Development, IDA=International Development Association, IDB=Inter-American Development Bank, IFAD=International Fund for Agricultural Development, OFID=OPEC Fund for International Development, UNAIDS=The Joint Programme on HIV/AIDS, UNDP=United Nations Development Program, UNECE=United Nations Economic Commission for Europe, UNFPA=United Nations Population Fund, UNICEF=United Nations Children's Fund, UNPBF=United Nations Peace Building Fund, UNRWA=United Nations Relief and Works Agency, WFP=United Nations World Food Programme, WHO=World Health Organization.
