# Supplementary material for: Donor funding health policy and systems research in low- and middle-income countries: how much, from where and to whom
Source: Health Res Policy Syst. 2017 Aug 31;15:68. doi: 10.1186/s12961-017-0224-6 (PMC5577666; doi:10.1186/s12961-017-0224-6)
Supplement: Supplementary file 2 — Alliance reports searched for keyword analysis. (PDF 35 kb) [file 12961_2017_224_MOESM2_ESM.pdf]

Appendix Table 2: Alliance reports searched for keyword analysis

Year	Author	Title	Available at:
2004	Alliance for Health Policy and Systems Research	Strengthening Health Systems: The Role and Promise of Policy and Systems Research	http://www.who.int/alliance-hpsr/resources/Strengthening_complet.pdf
2007	Green A, Bennett S, and the Alliance for Health Policy and Systems Research	Sound Choices: Enhancing Capacity for Evidence-Informed Health Policy	http://www.who.int/alliance-hpsr/resources/publications/9789241595902/en/
2009	Savigny D, Adam, T, and the Alliance for Health Policy and Systems Research	Systems Thinking for Health Systems Strengthening	http://www.who.int/alliance-hpsr/resources/flagshipreports/en/
2014	Bigdeli M, Peters D, Wagner A, and the Alliance for Health Policy and Systems Research	Medicines in Health Systems: Advancing access, affordability and appropriate use	http://www.who.int/alliance-hpsr/resources/publications/9789241507622/en/
2012	Gilson L and the Alliance for Health Policy and Systems Research	Health Policy and Systems Research - A Methodology Reader	http://www.who.int/alliance-hpsr/resources/publications/9789241503136/en/
2013	Peters D, Tran N, and Adam T	Implementation Research in Health: A Practical Guide	http://www.who.int/alliance-hpsr/resources/implementationresearchguide/en/
2012	World Health Organization	Changing Mindsets - The WHO Strategy on Health Policy and Systems Research	http://www.who.int/alliance-hpsr/resources/publications/9789241504409/en/
