# Supplementary material for: Donor funding health policy and systems research in low- and middle-income countries: how much, from where and to whom
Source: Health Res Policy Syst. 2017 Aug 31;15:68. doi: 10.1186/s12961-017-0224-6 (PMC5577666; doi:10.1186/s12961-017-0224-6)
Supplement: Supplementary file 3 — List of keywords used in analysis. (PDF 39 kb) [file 12961_2017_224_MOESM3_ESM.pdf]

Appendix Table 3: List of keywords used in analysis

Health Policy and Health Systems			Research	Flag Words
**1** access	**55** healthcare system	**110** public hospital	**1** action research	**1** immigration
**2** advancing access	**56** hospital	**111** public sector	**2** analysis	**2** development policy
**3** affordability	**57** hospital care	**112** public spending	**3** assess	**3** poverty reduction
**4** budget support	**58** hospital management	**113** quality	**4** audit	**4** nutrition
**5** capacity building	**59** human resource	**114** quality improvement	**5** case studies	**5** sanitation
**6** clinic	**60** human resources for health	**115** reform	**6** case study	
**7** cost effective	**61** immunization	**116** reform process	**7** causal	
**8** costs	**62** impact	**117** report	**8** cost effective	
**9** data	**63** implementation	**118** sector reform	**9** costs	
**10** decision maker	**64** influence	**119** service delivery	**10** data	
**11** decision making	**65** information	**120** social capital	**11** data collection	
**12** delivery	**66** information system	**121** social determinants	**12** economic	
**13** doctor	**67** infrastructure	**122** social determinants of health	**13** effectiveness	
**14** drug	**68** insurance scheme	**123** social network	**14** equity	
**15** economic	**69** intellectual property	**124** social science	**15** evaluate	
**16** effective	**70** knowledge	**125** social scientist	**16** evaluation	
**17** equitable	**71** leadership	**126** socio economic	**17** evidence	
**18** equity	**72** medical care	**127** staff	**18** focus group	
**19** essential medicines	**73** medical centre	**128** statistics	**19** group discussion	
**20** financial incentive	**74** medical education	**129** strengthen health systems	**20** health research	
**21** financial incentives	**75** medicine	**130** strengthening health systems	**21** health systems approach	
**22** financial sustainability	**76** national health insurance	**131** system performance	**22** health systems interventions	
**23** financing	**77** network	**132** system strengthening	**23** impact	
**24** guideline	**78** network development	**133** systems	**24** influence	
**25** guidelines	**79** nurses	**134** systems approach	**25** intervention	
**26** health care	**80** p4p	**135** systems intervention	**26** interview	
**27** health centre	**81** performance	**136** systems perspective	**27** investigate	
**28** health coverage	**82** personnel	**137** systems research	**28** method	
**29** health equity	**83** pharmaceutical	**138** systems strengthening	**29** mixed method	
**30** health facilities	**84** policies	**139** systems thinking	**30** performance	
**31** health financing	**85** policy	**140** theory	**31** policy analysis	
**32** health information	**86** policy analysis	**141** training	**32** policy intervention	
**33** health information system	**87** policy change	**142** trust	**33** process	
**34** health insurance	**88** policy development	**143** uhc	**34** process evaluation	
**35** health market	**89** policy implementation	**144** universal health	**35** qualitative	
**36** health organisation	**90** policy intervention	**145** universal health coverage	**36** qualitative research	
**37** health planning	**91** policy maker	**146** vaccine	**37** question	
**38** health planning and management	**92** policy process		**38** report	
**39** health policies	**93** policymaker		**39** research	
**40** health policy	**94** political		**40** research method	
**41** health policy and planning	**95** politics		**41** research question	
**42** health policy and systems	**96** primary care		**42** research strategy	
**43** health policy and systems research	**97** primary health		**43** researcher	
**44** health professionals	**98** primary health care		**44** respondent	
**45** health project	**99** private health sector		**45** social science	
**46** health reform	**100** private hospital		**46** statistics	
**47** health sector	**101** private sector		**47** study	
**48** health sector reform	**102** process		**48** study design	
**49** health services	**103** process evaluation		**49** survey	
**50** health spending	**104** provision		**50** systems interventions	
**51** health system	**105** provision		**51** systems research	
**52** health system strengthening	**106** public health		**52** systems thinking	
**53** health worker	**107** public health policy		**53** theory	
**54** healthcare	**108** public healthcare		**54** understand	
