# Supplementary material for: Donor funding health policy and systems research in low- and middle-income countries: how much, from where and to whom
Source: Health Res Policy Syst. 2017 Aug 31;15:68. doi: 10.1186/s12961-017-0224-6 (PMC5577666; doi:10.1186/s12961-017-0224-6)
Supplement: Supplementary file 4 — Donor-specific assumptions for in-depth project review. (PDF 40 kb) [file 12961_2017_224_MOESM4_ESM.pdf]

Appendix Table 4: Donor Specific Assumptions for in-depth project review

Donor type	Assumptions made
Multilateral Donors	World Bank's project database contains data on IBRD and IDA commitments for each loan. The Bank provides a breakdown of the percentage of project funds allocated to different sectors and themes. We examined individual project documents for the 20 largest IBRD projects (59% of total IBRD commitments) and the 20 largest IDA projects (40% of total IDA commitments) and adjusted project commitments to reflect the percentage of project funds allocated to health systems performance. On average, 26% of IBRD and 24% of IDA project funds were allocated to health systems performance. Based on these averages, we allocated 25% of commitments from multilateral donors to HPHS and 5% to HPSR.
Bilateral Donors	We estimated bilateral commitments to HPHS and HPSR based on information obtained from four agencies (USAID, AusAID, DFID, and CIDA). The funding assumptions applied to USAID are based on estimates of annual funding in target health areas (Malaria, Nutrition, HIV/AIDS, Maternal and Child Health, Service Delivery, Family Planning and Reproductive Health, Tuberculosis, Other Public Health Threats, Health Systems Strengthening, Program Design and Learning, and Administration and Oversight) provided in two USAID Reports to Congress on health-related research and development activities. The reports cover the period 2006-2013. Detailed information on projects funded by CIDA from 1998-2013 are available online through the Government of Canada International Development Project Browser. We reviewed project profiles for the top 20 projects funded by CIDA, which include a breakdown of the percentage of project funds allocated to different sectors and themes and calculated an average percentage allocated to HPHS and HPSR<br>We reviewed annual budget portfolios for DFID and AusAid to calculate the average percentage of health aid allocated to HPHS and HPSR for the years 2006-2012. For DFID, it is important to note that total commitments for HPSR over the period 2006-2010 are substantially lower than disbursements for HPSR—$84 million in commitments compared to $157 million in disbursements—and therefore it is important to take this into consideration when interpreting these estimates. Based on the averages from USAID, AusAID, DFID, and CIDA, we allocated 25% of commitments from bilateral donors to HPHS and 5% to HPSR.
The Global Fund	The grants database made available online by GFATM provides on project summary budgets, which include a breakdown of funding by expenditure category. We reviewed the budgets of the top 31 Global Fund projects (25% of all Global Fund projects) and calculated the average percentage allocated to HPHS and HPSR across projects. We allocated 15% of Global Fund commitments to HPHS and 5% to HPSR.
GAVI	GAVI's transparency portal includes information on Gavi's commitments and disbursements by type of support, country and year for the years 2005-2014. We compared annual commitment amounts for health systems strengthening in the portal to those in CRS database to confirm that projects designated as HPHS were categorized correctly.
Bill and Melinda Gates Foundation	To estimate HPHS and HPSR commitments from the Gates Foundation, we reviewed the descriptions of the largest 4 projects within each CRS subsector (CRS subsectors are described in the chart below) to determine if projects were relevant to HPHS and HPSR. We found that 50% of projects reviewed involved HPHS activities and roughly 25% of projects involved HPSR activities.
UN Agencies	Information on HPSR funding among UNICEF, UNDP, UNFPA proved difficult to obtain. We therefore employed the same method used for the Gates Foundation to estimate HPHS and HPSR commitments. We reviewed the descriptions of the largest 4 projects within each CRS subsector (CRS subsectors are described in the chart below) to determine if projects were relevant to HPHS and HPSR. We allocated 25% of commitments from UN Agencies to HPHS and 5% to HPSR.
